# Characterization of Esophageal Cancer and Its Association with Influencing Factors in Guangzhou City, China

**DOI:** 10.3390/ijerph17051498

**Published:** 2020-02-26

**Authors:** Cheng Cui, Hang Dong, Hongyan Ren, Guozhen Lin, Lu Zhao

**Affiliations:** 1 State Key Laboratory of Resources and Environmental Information System, Institute of Geographic Sciences and Natural Resources Research, Chinese Academy of Sciences, Beijing 100101, China; cuic@lreis.ac.cn (C.C.); zhaolu18@mails.ucas.ac.cn (L.Z.); 2 College of Resources and Environment, University of Chinese Academy of Sciences, Beijing 100190, China; 3 Department of Biostatistics and Cancer Registration, Guangzhou Center for Disease Control and Prevention, Guangzhou 510440, China; donghang83857490@126.com

**Keywords:** esophageal cancer, spatial variation, socioeconomic conditions, medical resources, ageing degree, Guangzhou city

## Abstract

Epidemiological features of esophageal cancer (EC), as well as their associations with potential influencing factors in a city, have seldom been seldom explored on a fine scale. The EC death cases in Guangzhou city during 2012−2017 were collected to describe the epidemiological characteristics such as EC mortality rate (ECMR) and health-seeking behaviors of deaths. Potential influencing factors, including socioeconomic conditions (population density, gross domestic product density), medical resources, and ageing degree were also gathered for exploring their relationships with the epidemiological characteristics of EC. A total of 2,409 EC deaths were reported during 2012−2017 in Guangzhou with an age-standardized ECMR of 3.18/10^5^. The prevalence of EC in Guangzhou was spatially featured and was divided into three regions with obvious differentiated ECMR (ECMR of 6.41/10^5^ in region A, ECMR of 5.51/10^5^ in region B, ECMR of 2.56/10^5^ in region C). The street/town-level ECMR was spatially clustered in Guangzhou city, especially two clusters of streets/towns with high ECMR were highlighted in region A and B respectively. Meanwhile, demographic features including gender gap, death age, temporal interval between diagnosis and death, health-seeking behaviors were remarkably different among the three regions. Moreover, health-seeking behaviors (e.g., the proportion of hospital deaths) of the EC deaths were obviously influenced by medical institution occupancy rate and socioeconomic conditions at street/town level. In addition, the street/town-level ECMR was significantly associated with ageing degree across Guangzhou city (r = 0.466, *p* < 0.01), especially in region A (r = 0.565, *p* < 0.01). In contrast, the ECMR in region B was closely related to population density (r = −0.524, *p* < 0.01) and gross domestic product density (r = −0.511, *p* < 0.01) when the ageing degree was controlled, while these associations were weak in region C. The epidemiological characteristics of EC in Guangzhou city were spatially featured and potentially associated with socioeconomic conditions, medical resources and ageing degree on a fine scale across Guangzhou city. This study could provide scientific basis for local authorities to implement more targeted EC interventions.

## 1. Introduction

Esophageal cancer (EC) is a worldwide fatal malignant tumor and causes about 0.5 million deaths in 2018 [1]. Accounting for approximately 50% of the global EC new cases and deaths, China is one of the countries with the highest EC incidence and mortality, and EC has been a serious health threat to the Chinese for decades [2,3,4].

As a common digestive tract malignant tumor, EC is closely associated with genetic, environmental, and behavioral risk factors [5,6,7]. The occurrence of EC increases along with aging [5], and the risk of developing EC is strongly associated with the positive family history of this disease [6]. Unsafe drinking water due to water pollution and insufficient drink water supply plays an important role in the EC occurrence [8,9]. Moreover, personal lifestyle choices, like smoking, heavy drinking, poor oral health, unhealthy diet (hot/salty/pickled food, low consumption of fruits and vegetables, etc.) also pose important effects on this disease [10,11,12,13,14,15]. In addition, evidences support that EC prevalence tend to be related with some socioeconomic conditions including the patients’ income level, occupation, and accessibility of medical resources [16,17,18,19].

It has been widely reported that the EC prevalence presents remarkable spatial differences at the continent, country, province, city, and county level, due to the geographical disparities of above influencing factors [1,4,7,17,20,21,22,23]. Meanwhile, the EC epidemic was also geographically featured by the urban-rural differences in China, including the EC mortality rate (ECMR) and their descending degrees in recent years [24,25,26,27,28]. As a whole, much attention has been consecutively paid to the rural areas with relatively higher ECMR while the epidemiological characteristics of EC in urban area was somewhat neglected in China for decades, in particular their internal differences inside a city remains unclear.

Guangzhou city is one of the biggest cities in the South China. This city went through a fast urbanization over the past decades, resulting in a highly urbanized population rate of 86.14% and a huge achievement of gross domestic product (GDP) with the GDP per capita exceeded $20,000 in 2017 [29]. Additionally, some issues in this city have attract much attention in recent years, including unbalanced development [30], uneven accessibility of medical resources [31], increasingly ageing population [32], and so on. However, the epidemiological characteristics of EC, as well as their associations with some potential influencing factors in this city, were seldom explored on a fine scale.

Therefore, the current study aimed to: (1) investigate the temporal and spatial patterns of epidemiological characteristics of EC in Guangzhou during 2012−2017, and to (2) explore the association between epidemiological characteristics of EC and potential influencing factors such socioeconomic conditions, medical resources and ageing degree across this city on a fine scale. This study would provide scientific basis for local authorities to implement more targeted EC interventions.

## 2. Materials and Methods

### 2.1. Study Area

**Figure 1:**
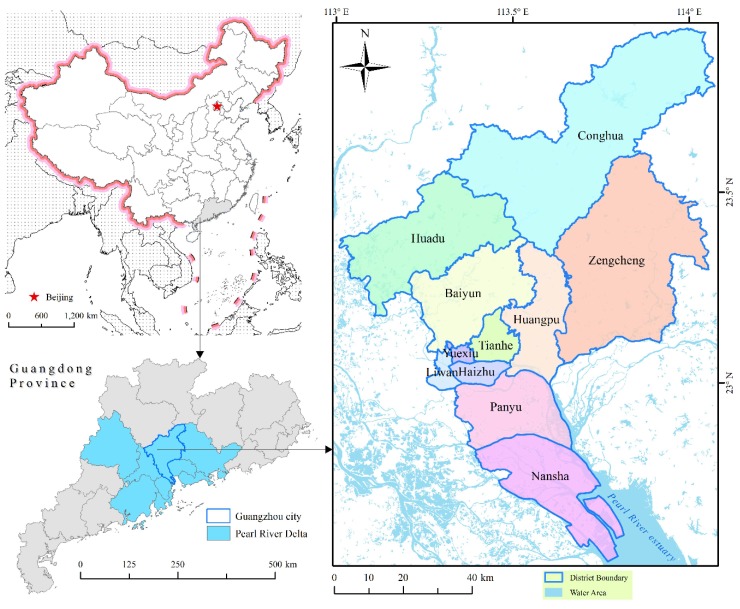
Illustration map of Guangzhou city.

Guangzhou city is located in the Pearl River Delta of Guangdong Province (Figure 1), which is one of the most developed areas in South China. It has a population of approximately 14.5 million permanent residents and covers an area of 7434 square kilometers with 11 districts comprising 170 streets/towns in total. This city is dominated by a subtropical monsoon climate with an average annual temperature of 21.5–22.2 °C and an average annual rainfall of over 1800 mm.

### 2.2. Data Collection and Processing

The EC death cases during 2012−2017 were collected from the death surveillance system of Guangzhou Center for Disease Control and Prevention. This dataset included each patient’s disease related demographic information (gender, age, and home address) and diagnostic information (the highest level of medical institution involved in the diagnosis, the temporal interval between diagnosis and death, and place of death). The patient’s address was geo-coded (restapi.amap.com/v3/geocode) so as to produce a spatial point layer.

Some variables which may have potential associations with epidemiological characteristics of EC were collected, including the population density, GDP density, medical resources, and ageing degree (Table 1). The gridded population density and GDP density data were disaggregated from the district level statistics data based on the land-use type, road density and nighttime light [33,34]. GDP density is a measure of economic activity by area and it reflects a place’s economic density and vitality. Both of the population density and GDP density were used to measure the regional socioeconomic conditions in Guangzhou city, which may affect family income, lifestyles and medical service level [35,36]. Since the ascending trend of total cancer incidence and mortality in Guangzhou attributed largely to the ageing population [37], the ageing degree of each street/town, in terms of the proportion of the elderly (over 65) population, was obtained from the Sixth National Population Census of the People’s Republic of China dataset.

Local medical institutions were categorized into three levels: (1) the top medical institutions (TMIs) i.e., the tertiary hospitals with the highest medical service in Guangzhou and even in South China, (2) middle medical institutions (MMIs) including hospitals at other grades, and (3) the basic medical institutions (BMIs) that provide health care services for local people. Across the whole city, the mean values of hospital beds in TMI, MMI, and BMI were respectively 985, 188, and 24 according to the statistics of Guangzhou Municipal Health Commission (wjw.gz.gov.cn). Moreover, the rate of medical institutions occupancy (RMIO) per 10,000, including RMIO of TMIs (RMIO_TMI_), MMIs (RMIO_MMI_), BMIs (RMIO_BMI_), and all medical institutions (RMIO_all_), as well as the number of hospital beds per 1000 people was employed to indicate the medical resources among 170 streets/towns. In addition, the capability of each street/town being covered by TMIs was further termed by the Euclidean distance between the centroids of the towns/streets and the nearest TMI.

### 2.3. Epidemiological and Statistical Analysis

The Guangzhou crude ECMR in each year was calculated based on population data from Guangzhou statistics bureau, and the crude ECMR was age-standardized based on Chinese standard population in 2000.

In order to depict the epidemiological characteristics of EC on a fine scale, 170 streets/towns in total were taken into account as the basic unit for statistical calculations. The average size and population of these streets/towns were 43.73 km^2^ and 85,294 people, respectively. The annual average crude ECMR was calculated for 170 streets/towns in Guangzhou based on the population density data in 2015 (Table 1), and the spatial empirical Bayes smoothing method (GeoDa 1.12, the Center for Spatial Data Science, Chicago, IL, USA) was applied to reduce the spatial instability of the street/town-level ECMR before statistical and spatial analysis.

The ratio of number of EC deaths to the corresponding unit’s area was calculated as the density of EC death cases. Additionally, the gender ratio, mean value of death age, mean/median temporal interval between diagnosis and death, the selection of medical institution for diagnosis, and the selection of death place were used to describe the epidemiological characteristics of EC.

The correlation between epidemiological characteristics and influencing factors was addressed by Pearson correlation coefficients and partial correlation analysis. These statistical analyses were performed using SPSS 19.0 (SPSS Inc., Chicago, IL, USA).

### 2.4. Spatial Analysis

In order to show the density distribution of EC death case during 2012−2017, the kernel density estimation was employed in this study. This method could produce a smooth density surface over space by calculating EC deaths intensity as density estimation [38]. Furthermore, the Gaussian mixture model was used to cluster these cases so as to identify the regional differences of this disease across the whole city [39]. The boundaries of each region were determined by the minimum convex polygon of each EC case cluster. Then each street/town was labeled with different region ID (A, B, C, etc.) based on the centroids of these streets/towns.

Meanwhile, Moran’s I was used to detect whether significant spatial autocorrelation of ECMR existed at street/town level across Guangzhou city. The Moran’s I was calculated as follows:

(1) $$I=\;\frac{n\sum_{i=1}^{n}\sum_{j=1}^{n}\omega_{ij}\left(x_{i}-\bar{x}\right)\left(x_{j}-\bar{x}\right)}{\sum_{i=1}^{n}\sum_{j=1}^{n}\omega_{ij}{\left(x_{i}-\bar{x}\right)}^{2}}$$

where n is the number of streets/towns; $x_{i}$ and $x_{j}$ stand for the ECMR of street/town i and j; $\bar{x}$ is the average value of ECMR; and $\omega_{ij}$ is the spatial weight between street/town i and j that can be defined by the contiguity of these streets/towns. The value of Moran’s I falls between −1 and 1 [40]. A statistically significant positive value of Moran’s I indicates the presence of ECMR spatial cluster, i.e., adjacent streets/towns had similar ECMR, whereas a statistically significant negative Moran’s I implied a tendency toward dispersal.

On the basis of the existence of spatial autocorrelation, the hotspot analysis was further utilized to capture the specific regions (streets/towns) with clustered high or low ECMRs in our study. We selected Getis-Ord Gi* to identify the statistically significant hot and cold spots according to the following formula [41,42]:

(2) $$G_{i}^{*}=\frac{\sum_{j=1}^{n}\omega_{ij}x_{j}-\bar{X}\sum_{j=1}^{n}\omega_{ij}}{S\sqrt{\frac{\left[n\sum_{j=1}^{n}\omega_{ij}{}^{2}-{\left(\sum_{j=1}^{n}\omega_{ij}\right)}^{2}\right]}{n-1}}}$$

where $x_{j}$ is the ECMR value for street/town j; $\omega_{ij}$ stands for the spatial weight between street/town i and j; n means the number of streets/towns; and:

(3) $$\bar{X}=\;\frac{\sum_{j=1}^{n}x_{j}}{n}$$

(4) $$S=\sqrt{\frac{\sum_{j=1}^{n}x_{j}{}^{2}}{n}-({\bar{X)}}^{2}}$$

The Gi* statistic is a Z-score; for statistically significant positive Z-scores, the larger the Z-score is, the more intense the clustering of high ECMR (i.e., a hot spot); for statistically significant negative Z-scores, the smaller the Z-score is, the more intense the clustering of low ECMR (i.e., a cold spot). In reality, the so-called hot spot is most useful for disease control and prevention. 

The Gaussian mixture model was achieved using the scikit-learn [43]. Other spatial analyses were completed in the ArcGIS 10.2 platform (ESRI, Redlands, CA, USA).

## 3. Results

### 3.1. Temporal and Spatial Patterns of the EC Prevalence

**Figure 2:**
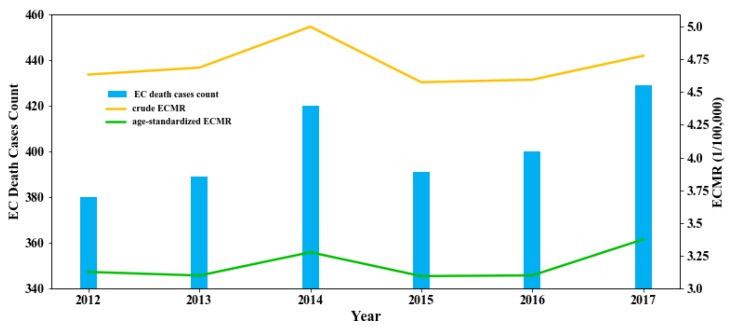
Temporal trends of EC prevalence in Guangzhou city.

A total of 2409 EC death cases were reported during 2012−2017 in Guangzhou city. The annual average crude and age-standardized ECMR were 4.71/10^5^ and 3.18/10^5^ respectively (Figure 2).

**Figure 3:**
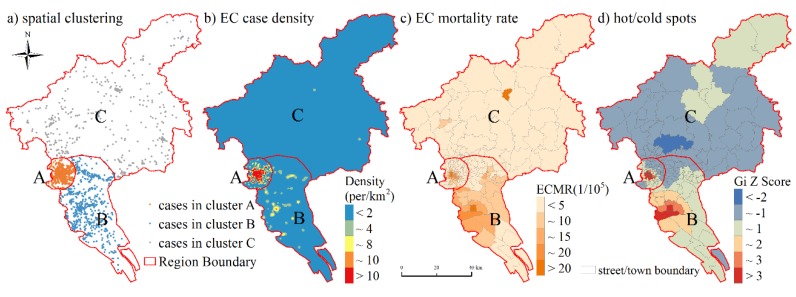
Spatial distribution of EC prevalence. (**a**) spatial clustering results achieved by the Gaussian mixture model. The minimum convex polygon of each EC case cluster was used to determine the boundary of Regions A, B, and C; Region A: mainly in central districts, including Liwan, Yuexiu, the west of Haizhu and Tianhe. Region B: Panyu, Nansha, the east of Haizhu and Tianhe, the south of Huangpu. Region C: Huadu, Conghua, Zengcheng, Baiyun, the north of Huangpu. (**b**) EC death case density distribution derived from kernel density estimation; (**c**) annual average crude ECMR in each street/town; (**d**) hot/cold spots of annual average ECMR.

Although the annual number of EC deaths presented a slightly ascending trend (Figure 2), the spatial distribution of EC deaths remained stable in each year (Figure S1). Most death cases were clustered in the central region of Guangzhou city, and the southern part was accompanied with a relatively higher density of EC deaths while the northern half with sparsely distributed EC death cases (Figure 3a,b). The spatial distribution of ECMR further indicated the obvious regional difference of EC prevalence (Figure 3c).

The boundaries of three distinguished regions were clearly identified across the city according to Gaussian mixture model (Figure 3). The streets/towns with relatively higher ECMR were mostly located in Region A or B (Figure 3c). In comparison, the lowest crude ECMR was observed in region C (Table 2). Global positive spatial autocorrelation pattern of ECMR (Moran’s I = 0.71, *p* < 0.01) was revealed by spatial autocorrelation analysis. Figure 3d further illustrated that two remarkable ECMR hotspots of higher Gi Z-score were respectively included in region A and region B, while region C was mainly covered by cold spots with lower Gi Z-score. It can be seen that the EC prevalence showed clearly regional difference and was spatially featured at street/town scale.

### 3.2. Demographic Features and Health-Seeking Behaviors of EC Death Cases

Except for spatially differentiated ECMRs among three regions in Guangzhou city, the regions of A, B, and C were also featured by the demographic feature difference of these deaths. As given in Table 2, approximately 87% of EC death cases occurred in man and the largest gender ratio was observed in region B (10.49:1).

The average age of EC deaths was 65.8 in the whole city with differences among three regions (A > C > B). The region with a higher mean value of EC death age was associated with a shorter temporal interval between diagnosis and death. More than half of these cases died within one year after their diagnosis according to the median value (9 months) of temporal interval in the whole city. These results showed that region A and B possessed different demographic features of this disease while region C owned moderate gender gap, death age, and temporal interval between diagnosis and death.

With regard to all EC deaths in Guangzhou, majority of the patients (67.66%, *n* = 1630) had been to a TMI to confirm EC (Table 3). In term of the death place, the proportion of hospital death (48.36%, *n* = 1165) and home death (48.73%, *n* = 1174) were almost equal.

Although TMI was the dominant choice to confirm EC for patients in the regions of A, B and C, the proportion of patients ever diagnosed in TMI had regional differences ranked as A (79.79%) > B (59.95%) > C (54.94%). Meanwhile, the regional differences were even larger in the selection of death place. The proportion of home death in region B (72.71%) and region C (72.92%) was obviously higher than that in Guangzhou city (48.73%), while this value in region A was merely 18.33%. It can be seen that health-seeking behavior of EC case presented clear regional variations in Guangzhou city.

### 3.3. Potential Influences of Socioeconomic Conditions on EC Epidemiological Features

**Figure 4:**
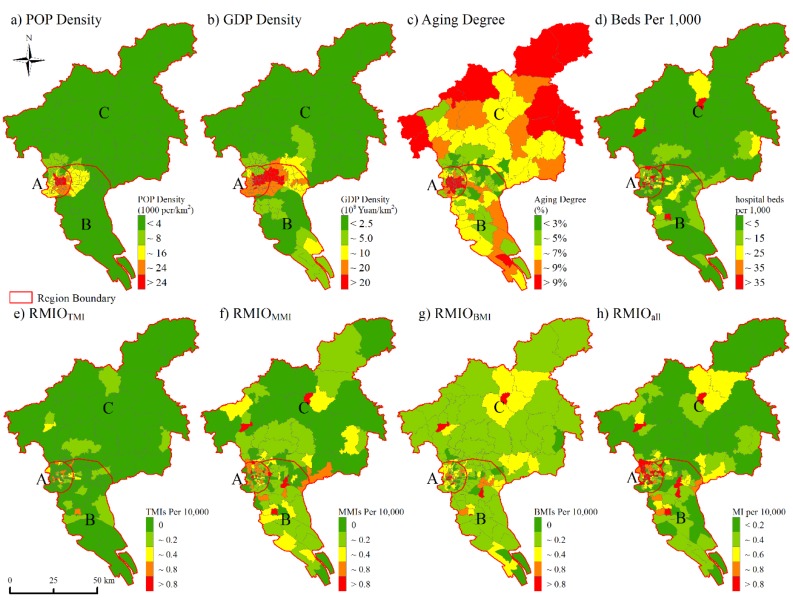
The spatial distribution of potential influencing factors in Guangzhou on street/town scale. (**a**) the population density; (**b**) the gross domestic products density; (**c**) the ageing degree; (**d**) hospital beds per 1000 people; (**e**–**h**) indicate the rate of medical institutions occupancy (RMIO) per 10,000, including top medical institutions(RMIO_TMI_), middle medical institutions (RMIO_MMI_), basic medical institutions (RMIO_BMI_) and all medical institutions (RMIO_all_).

The streets/towns with dense population and advanced economic level were mainly distributed in region A and region B (Figure 4a,b). Higher ageing degree (>9%) were observed in some central streets, as well as some outer towns (Figure 4c). Meanwhile, the streets in region A possessed higher values of RMIO_TMI_ and hospital beds per 1000 (Figure 4d,e). In comparison, the occupancy of MMIs and BMIs presented relatively sparse distribution (Figure 4f,g). Table S1 further showed the regional differences of these influencing factors.

In addition, there was a strong correlation between population density and GDP density (r = 0.891, *p* < 0.01) at the street/town level (Table 4). The streets/towns with higher GDP density or population density possessed many more medical resources, e.g., hospital beds (r = 0.236, *p* < 0.01; r = 0.264, *p* < 0.01) and TMIs (r = 0.260, *p* < 0.01; r = 0.313, *p* < 0.01). And the TMIs were easier to be visited by the residents in these streets/towns due to shorter distance from the centroids of them to the nearest TMI (r = −0.376, *p* < 0.01; r = −0.480, *p* < 0.01). These results showed that streets/towns with higher level of socioeconomic conditions had a better access of medical resources.

In general, streets in region A were featured by a dense population and well-developed economy, and easy access to high quality medical services, while the highest ageing degree was also observed in region A. The epidemiological features of EC were spatially featured, especially the health-seeking behaviors and ECMR. There may be potential associations between them and these spatially differentiated influencing factors.

According to Table 4, the low proportion of EC deaths visiting TMI to confirm EC (PMID_TMI_) was significantly correlated with low GDP density (r = 0.313, *p* < 0.01), long distance to a TMI (r = −0.444, *p* < 0.01), and low RMIO_TMI_ (r = 0.187, *p* < 0.05) on street/town scale. Moreover, the proportion of hospital death was associated with socioeconomic conditions and access to health care system, including GDP (r = 0.541, *p* < 0.01), hospital beds per 1000 (r = 0.310, *p* < 0.01) and RMIO_all_ (r = 0.256, *p* < 0.01). These results showed that socioeconomic conditions and accessibility of medical resources were important factors in determining the health-seeking behaviors of EC deaths in Guangzhou.

The positive correlation between ECMR and ageing degree was observed (r = 0.466, *p* < 0.01) on the street/town scale across Guangzhou city (Table 5). Since collinear relationship existed between ageing degree and other variables (Table 4), partial correlation analysis showed that the population density (r = −0.413, *p* = 0.064) and GDP density (r = −0.168, *p* < 0.05) was negatively correlated with ECMR when the ageing degree was controlled.

In addition, there were obvious differences in the correlation between ECMR and potential influencing factors within regions of A, B, and C. The ECMR in region A was weakly correlated with population density (r = −0.105, *p* = 0.419) and GDP density (r = −0.132, *p* = 0.312), and was strongly correlated with ageing degree (r = 0.565, *p* < 0.01). However, significant negative coefficients were found between ECMR and population density (r = −0.470, *p* < 0.01) and GDP density (r = −0.426, *p* < 0.01) after the control of ageing degree. In comparison, these correlation coefficients only showed slightly fluctuation in region B and C when ageing degree was controlled. This was because of the weak correlations between ECMR and ageing degree (region B: r = 0.173, *p* = 0.211; region C: r = 0.003, *p* = 0.982). Population density (r = −0.524, *p* < 0.01) and GDP density (r = −0.511, *p* < 0.01) posed significant effects on ECMR in region B, while these associations were relatively weak in region C. The street/town-level ECMR tended to be closely associated with socioeconomic conditions and ageing degree to various degree inside the whole city and regions A, B and C. The ageing degree was an important influence factor of the ECMR spatial variations in the whole city and region A, while the ECMR in region B was closely associated with population density and GDP density.

## 4. Discussion

Our study conducted an investigation on the spatial variations of epidemiological features of EC in Guangzhou city, and explored their associations with potential influencing factors on the street/town scale. Several interesting findings were achieved and would help local authorities recognize the prevalence of this disease and taking more effective measures to reduce its threat to the public health in Guangzhou city.

In this study, both crude and age-standardized ECMR in Guangzhou were far lower than the whole national average values in 2015 (13.68/10^5^ for crude ECMR and 8.33/10^5^ for age-standardized ECMR respectively), and lower than the city average level (9.99/10^5^ for crude ECMR and 5.87/10^5^ for age-standardized ECMR respectively) [24]. And this gap was consist with previous reports [2,44]. We think this gap was probably associated with the great achievements and lifestyle changes related to the socioeconomic developments in Guangzhou, such as a healthy diet (lower intake of salt, increasing consumption of fruits and vegetables, etc.), safe drinking water, great awareness of health issues and better access to healthcare system all reduced the risk of developing EC [27,45,46,47,48,49].

However, the street/town ECMR was spatially clustered in Guangzhou city, especially two hotspots were highlighted in region A and B respectively. ECMR hotspot in region A was closely associated with its high ageing degree, and relatively low socioeconomic condition posed an effect on ECMR in region B. To the best of our knowledge, this is the first comprehensive study of epidemiological characteristics of EC in Guangzhou city up to now.

The spatially differentiated epidemiological characteristics of EC were also featured by the health-seeking behaviors of EC deaths, including the selection of hospital to confirm EC and the death place. Most patients chose TMI to confirm EC for EC is one of the most health-threating diseases, however, this proportion (PMID_TMI_) was affected by the regional socioeconomic conditions and accessibility of medical resources. And this association was stronger for the selection of death place. Some studies have shown that socioeconomic status may be involved in the decision making of esophageal cancer treatment [19,50]. And we cautiously suggest the health care system should be further completed to address the barriers to health-seeking behaviors of EC patients in Guangzhou city.

A preliminary trial was conducted in this study to characterize the epidemiological features of EC, and to evaluate their relations to some potential influencing factors. However, due to the complicated process from the onset to the death of EC case, it is necessary to combine the information of both EC incidence and deaths to deeper clarify the specific mechanism of these associations. Our present study showed the spatially differentiated epidemiological characteristics of EC were closely associated with socioeconomic conditions, medical resources and ageing degree. The present results showed that ageing degree was one important factor of ECMR variations in Guangzhou especially in region A. According to that, we cautiously suggest that the interventions should be strengthened and focus on the elderly especially in region A, such as early screening program, which could reduce the prevalence of EC effectively [51]. And region A with advanced socioeconomic conditions could provide financial supports for these interventions.

There were some limitations to this study. First, the detailed information about these deaths, especially the retrospective investigations, should be collected and analyzed to complete the study of associations between EC epidemiological features and socioeconomic conditions. Second, the period of included EC deaths was relatively short, a longer time interval of EC cases could help us further analyze the temporal and spatial patterns of EC epidemics in more detail in Guangzhou. Despite these limitations, these findings were insightful and of practical importance to implement effective interventions to reduce EC threat in Guangzhou.

## 5. Conclusions

In summary, the epidemiological characteristics of EC in Guangzhou city were spatially featured and potentially associated with medical resources, ageing degree and socioeconomic conditions on the street/town scale. We suggest the interventions should focus on the elderly especially in region A.

## Figures and Tables

**Table 1:** Overview of the influencing factors and data source.

Influencing Factors	Dataset	Time	Resolution	Source
Population density	Gridded population density	2015	1 km	www.resdc.cn
GDP density	Gridded GDP density	2015	1 km	
Medical resources	Medical institutions	2016	Vector (point)	data.gz.gov.cn; wjw.gz.gov.cn
Ageing degree	The Sixth National Population Census	2010	Street/town	www.stats.gov.cn

**Table 2:** Comparison of EC characteristics among regions of A, B, and C and Guangzhou city.

Region	% of EC Cases	Density (Cases/km^2^)	Crude ECMR (1/10^5^)	Gender Ratio (Male to Female)	Mean Value of Death Age (Year)	Mean/Median Value of Temporal Interval between Diagnosis and Death (Month)
Region A	44.17%	4.69	6.41	5.06:1	67.4	13.5/8.0
Region B	34.83%	0.55	5.51	10.49:1	63.9	15.7/12.0
Region C	21.00%	0.09	2.56	5.85:1	65.9	13.9/8.0
Guangzhou city	100%	0.33	4.71	6.46:1	65.8	14.4/9.0

**Table 3:** The highest level of medical institution for diagnosis and the place of death among the EC patients in Guangzhou city and region A, B, C.

Region		Death_Hosp	Death_Home	Death_Other	Death_Total
Guangzhou city	Diag_TMI	903	689	38	1630 (67.66%)
	Diag_MMI	192	254	17	463 (19.22%)
	Diag_BMI	68	216	14	298 (12.37%)
	Diag_Other	2	15	1	18 (0.75%)
	Diag_Total	1165 (48.36%)	1174 (48.73%)	70 (2.91%)	2409 (100%)
Region A	Diag_TMI	681	141	27	849(79.79%)
	Diag_MMI	127	27	11	165 (15.51%)
	Diag_BMI	16	24	6	46 (4.32%)
	Diag_Other	1	3	0	4 (0.38%)
	Diag_Total	825 (77.54%)	195 (18.33%)	44 (4.14%)	1064 (100%)
Region B	Diag_TMI	136	363	4	503 (59.95%)
	Diag_MMI	41	96	4	141 (16.81%)
	Diag_BMI	37	139	5	181 (21.57%)
	Diag_Other	1	12	1	14 (1.67%)
	Diag_Total	215 (25.63%)	610 (72.71%)	14 (1.67%)	839 (100.00%)
Region C	Diag_TMI	86	185	7	278 (54.94%)
	Diag_MMI	24	131	2	157 (31.03%)
	Diag_BMI	15	53	3	71 (14.03%)
	Diag_Other	0	0	0	0(0.00%)
	Diag_Total	125 (24.70%)	369 (72.92%)	12 (2.37%)	506 (100.00%)

Note: Death_Hosp, Death_Home, and Death_Other indicates the place of death case in hospital, at home, and other places including on the way to various medical institutions. Diag_TMI, Diag_MMI, Diag_BMI, and Diag_Other represents diagnosed in a TMI, MMI, BMI, and other medical institutions. Death_Total and Diag_Total respectively mean the total numbers (proportions) of death places and diagnosis selections of included EC death cases in Guangzhou, region A, region B, and region C.

**Table 4:** Correlation among influencing factors and health-seeking behaviors of EC cases in Guangzhou city during 2012–2017 (*N* = 170).

	POP	GDP	DistTMI	Beds	RMIOTMI	RMIOMMI	RMIOBMI	RMIOall
Ageing	0.557 **	0.480 **	−0.084	0.324 **	0.311 **	0.138	0.291 **	0.279 **
POP	1	0.891 **	−0.480 **	0.264 **	0.313 **	0.023	0.118	0.131
GDP		1	−0.376 **	0.236 **	0.260 **	0.050	0.177 *	0.161 *
DistTMI			1	−0.332 **	−0.284**	−0.204 **	−0.232 **	−0.296 **
Beds				1	0.870**	0.610 **	0.412 **	0.776 **
RMIOTMI					1	0.140	0.216 **	0.401 **
RMIOMMI						1	0.423 **	0.893 **
RMIOBMI							1	0.738 **
RMIOall								1
PMIDTMI	0.387 **	0.313 **	−0.444 **	0.178 *	0.187*	0.058	0.036	0.095
PMIDMMI	−0.193 *	−0.200 **	0.371 **	−0.082	−0.099	−0.002	−0.072	−0.052
PMIDBMI	−0.320 **	−0.210 **	0.230 **	−0.151 *	−0.147	−0.073	0.027	−0.072
PMIDother	−0.110	−0.071	0.050	−0.035	−0.043	−0.003	0.010	−0.008
PDPhosp	0.640 **	0.541 **	−0.487 **	0.310 **	0.284**	0.170*	0.194 *	0.256 **
PDPhome	−0.644 **	−0.554 **	0.521 **	−0.311 **	−0.281**	−0.166*	−0.198 **	−0.255 **
PDPother	0.028	0.083	−0.137	−0.001	0.001	−0.007	0.035	0.010

Note: Ageing: ageing degree; POP: population density; GDP: gross domestic product density; Dist_TMI_: distance between the centroids of the towns/streets and the nearest TMI. Beds: hospital beds per 1000; RMIO_TMI_: rate of medical institutions occupancy (RMIO) per 10,000 for top medical institutions; RMIO_MMI_: RMIO for middle medical institutions; RMIO_BMI_: RMIO for basic medical institutions; RMIOall: RMIO for all medical institutions; PMID: Proportion of the highest level of Medical Institution involved in the Diagnosis, including TMI (PMID_TMI_), MMI (PMID_MMI_), BMI (PMID_BMI_) and other medical institution (PMID_other_); PDP: Proportion of Death Place, including hospital (PDP_hosp_), home (PDP_home_), other (PDP_other_). ** Correlation is statistically significant at 0.01 level. * Correlation is statistically significant at 0.05 level.

**Table 5:** Correlation coefficients between ECMR and influencing factors on street/town scale.

Region	POP	GDP	Ageing
Guangzhou City (*N* = 170)	0.155 *	0.093 (*p* = 0.227)	0.466 **
Control Ageing	−0.143 (*p* = 0.064)	−0.168 * (*p* = 0.029)	/
Region A (*N* = 61)	−0.105 (*p* = 0.419)	−0.132 (*p* = 0.312)	0.565 **
Control Ageing	−0.470 **	−0.426**	/
Region B (*N* = 54)	−0.532 **	−0.529 **	0.173 (*p* = 0.211)
Control Ageing	−0.524 **	−0.511 **	/
Region C (*N* = 55)	−0.140 (*p* = 0.308)	−0.105 (*p* = 0.448)	0.003 (*p* = 0.982)
Control Ageing	−0.190 (*p* = 0.169)	−0.127 (*p* = 0.359)	/

Note: POP: population density. GDP: gross domestic product density; Ageing: ageing degree. ** Correlation is statistically significant at 0.01 level. * Correlation is statistically significant at 0.05 level.

## Supplementary Materials

The following are available online at https://www.mdpi.com/1660-4601/17/5/1498/s1, Figure S1: Spatial distribution of EC death cases in each year during 2012−2017. Table S1: The average, the standard deviation and the coefficient of variation of influencing factors in Guangzhou city and region A, B and C on street/town scale.

## Abbreviations

EC	Esophageal Cancer
ECMR	Esophageal Cancer Mortality Rate
TMI	Top Medical Institution
MMI	Middle Medical Institution
BMI	Basic Medical Institution
RMIO	The Rate of Medical Institutions Occupancy per 10,000
PMID	Proportion of the highest level of Medical Institution involved in the Diagnosis
PDP	Proportion of Death Place
