# Supplementary material for: Characterization of Esophageal Cancer and Its Association with Influencing Factors in Guangzhou City, China
Source: Int J Environ Res Public Health. 2020 Feb 26;17(5):1498. doi: 10.3390/ijerph17051498 (PMC7084651; doi:10.3390/ijerph17051498)
Supplement: Supplementary file 1 [file ijerph-17-01498-s001.pdf]

International Journal of
Environmental Research and Public Health

Supplementary Material:

Table S1 showed the unbalanced development in Guangzhou city for all of the coefficients of variation of influencing factors were greater than 100% except for the aging degree. As far as the medical resources were concerned, almost all of the coefficients of variation of relevant indicators were greater than 100% even in region A, B and C. The health care system was not equally accessible across Guangzhou city.

Table S1. The average, the standard deviation and the coefficient of variation of influencing factors in Guangzhou city and region A, B and C on street/town scale.

Region	POP (10^3per/km^2)	GDP (10^8yuan/km^2)	Aging degree	Beds	$RMIO_{TMI}$	$RMIO_{MMI}$	$RMIO_{BMI}$	$RMIO_{all}$
Region A (N=61)	21.54±10.70 (49.67%)	36.90±30.51 (82.69%)	10.48%±3.18% (30.34%)	21.18±24.63 (116.29%)	0.14±0.22 (157.14%)	0.37±0.49 (132.43%)	0.34±0.36 (105.88%)	0.85±0.79 (92.94%)
Region B (N=54)	7.24±6.58 (90.94%)	11.88±11.14 (93.73%)	5.01%±2.23% (44.51%)	8.12±11.86 (146.06%)	0.04±0.09 (225.00%)	0.20±0.27 (135.00%)	0.20±0.27 (135.00%)	0.43±0.44 (102.32%)
Region C (N=55)	2.53±2.27 (89.52%)	2.98±3.37 (113.13%)	5.66%± 2.57% (45.41%)	8.93±17.67 (197.87%)	0.04±0.10 (250.00%)	0.24±0.68 (283.33%)	0.17±0.17 (100.00%)	0.45±0.84 (186.67%)
Guangzhou city (N=170)	10.85±11.12 (102.46%)	17.98±24.30 (135.12%)	7.18%±3.67% (51.11%)	13.07±20.00 (153.02%)	0.07±0.16 (228.57%)	0.28±0.51 (182.14%)	0.24±0.29 (120.83%)	0.59±0.74 (125.42%)

Note: POP: population density; GDP: gross domestic product density; Beds: hospital beds per 1,000; $RMIO_{TMI}$: rate of medical institutions occupancy (RMIO) per 10,000 for top medical institutions; $RMIO_{MMI}$: RMIO for middle medical institutions; $RMIO_{BMI}$: RMIO for basic medical institutions; $RMIO_{all}$: RMIO for all medical institutions. The values in each grid was shown as average ± standard deviation (coefficient of variation). $Coeffficient\ of\ variations = \frac{\text{standard deviation}}{\text{average}} * 100\%$. The variable shows strong variations when its coefficient of variation is greater than 100%.

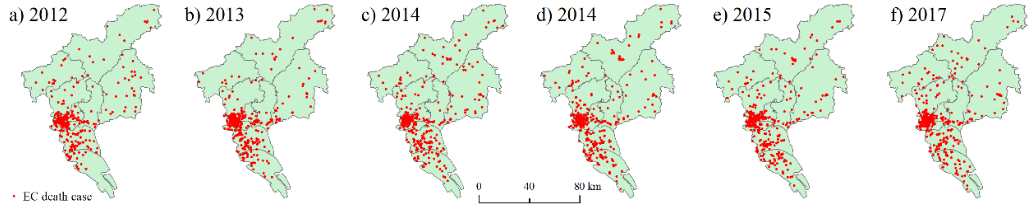

Figure S1. Spatial distribution of EC death cases in each year during 2012-2017.
